# Influence of sulfur and cadmium on antioxidants, phytochelatins and growth in Indian mustard

**DOI:** 10.1093/aobpla/plv001

**Published:** 2015-01-12

**Authors:** Humayra Bashir, Mohamed M. Ibrahim, Rita Bagheri, Javed Ahmad, Ibrahim A. Arif, M. Affan Baig, M. Irfan Qureshi

**Affiliations:** 1 Proteomics and Bioinformatics Lab, Department of Biotechnology, Jamia Millia Islamia, New Delhi 110025, India; 2 Department of Botany and Microbiology, Science College, King Saud University, PO Box 2455, Riyadh, Saudi Arabia; 3 Department of Botany and Microbiology, Faculty of Science, Alexandria University, PO Box 21511, Alexandria, Egypt

**Keywords:** Antioxidants, cadmium, growth, oxidative stress, phytochelatins, sulfur

## Abstract

Soils in many parts of the world are contaminated with heavy metals, leading to multiple, deleterious effects on plants and threats to world food production efficiency. Cadmium (Cd) is one such metal, being toxic at relatively low concentrations as it is readily absorbed and translocated in plants. Sulfur-rich compounds are critical to the impact of Cd toxicity, enabling plants to increase their cellular defence and/or sequester Cd into vacuoles mediated by phytochelatins (PCs). The influence of sulfur on Cd-induced stress was studied in the hyperaccumulator plant Indian mustard (*Brassica juncea)* using two sulfur concentrations (+S, 300 µM $\text{S}{{\text{O}}_{4}}^{2-}$ and S-deficient −S, $30\;\mu M\;\text{S}{{\text{O}}_{4}}^{2-}$) with and without the addition of Cd (100 µM CdCl_2_) at two different time intervals (7 and 14 days after treatment). Compared with control plants (+S/−Cd), levels of oxidative stress were higher in S-deficient (−S/−Cd) plants, and greatest in S-deficient Cd-treated (−S/+Cd) plants. However, additional S (+S/+Cd) helped plants cope with oxidative stress. Superoxide dismutase emerged as a key player against Cd stress under both −S and +S conditions. The activity of ascorbate peroxidase, glutathione reductase and catalase declined in Cd-treated and S-deficient plants, but was up-regulated in the presence of sulfur. Sulfur deficiency mediated a decrease in ascorbate and glutathione (GSH) content but changes in ascorbate (reduced : oxidized) and GSH (reduced : oxidized) ratios were alleviated by sulfur. Our data clearly indicate that a sulfur pool is needed for synthesis of GSH, non-protein thiols and PCs and is also important for growth. Sulfur-based defence mechanisms and the cellular antioxidant pathway, which are critical for tolerance and growth, collapsed as a result of a decline in the sulfur pool.

## Abstract (precis)

Sulphur(S)-deficiency is emerging as a major problem for agricultural productivity. Cadmium (Cd) exerts its phytotoxicity against defence, growth and development. S-rich compounds (glutathione, phytochelatins, etc.) limit the impacts of Cd-toxicity. We investigated what happens during S-deficiency and Cd exposure (dual stress) in mustard. Major findings were: S-deficiency increases the susceptibility of plants to Cd-generated oxidative damage and modulates the AsA-GSH antioxidant cycle; SOD is not the first line of defence against metal stress and S-rich metabolites play a prime role; S-deprived plants are more prone to Cd and oxidative stress; and great loss is incurred to defence modules and growth under dual stress, restricting the efficiency of phytoremediation.

## Introduction

In recent years, sulfur deficiency has become a major problem for agricultural productivity, reducing both crop quality and yields. Legitimate approaches to reducing emissions of sulfur into the atmosphere have resulted in a concomitant decrease in atmospheric deposition of sulfur on agricultural land. Based on crop demand, fertilizer-use efficiency and current inputs, the worldwide sulfur-deficit has been estimated to be 10.4 million tonnes annually (Haneklaus *et al*. 2007). Increased food production will further raise sulfur requirements, elevating this sulfur-deficit to 12.5 million tonnes by 2015 (Randazzo 2009).

As an essential macronutrient required for the proper growth and development of plants, sulfur plays a critical role in a number of cellular processes, such as in protein disulfide bridges (Saito 2000), mediating electron transport in iron–sulfur (Fe–S) clusters, the redox cycle, detoxification of heavy metals and xenobiotics, vitamin co-factors (Hell and Hillebrand 2001) and the metabolism of secondary products (Hell 1997; Saito 2000). Sulfur is thus a major plant nutrient (Takahashi *et al*. 1997; Leustek *et al*. 2000; Saito 2000; Lee and Korban 2002) that contributes to an increase in crop yield, directly adding nutritional value and improving the efficiency of use of other essential plant nutrients (Salvagiotti *et al*. 2009), particularly nitrogen and phosphorus. Research on plant adaptations to S-related stresses has shifted from an emphasis on excessive inputs and acidification to how deficiencies impact crop production. Sulfur deficiency not only impacts crop quality and yield, but it also raises the demand for adequate fertilization to resist biotic and abiotic stresses. Exposure of plants to excessive toxic metals like cadmium (Cd) may affect the uptake and metabolism of S and negatively impact the yield and plant resistance to abiotic stresses (Gill and Tuteja 2011).

Cadmium is a non-essential heavy metal that is readily absorbed and rapidly translocated in plants, which makes it highly bio-available and thus toxic, even at relatively low concentrations. Cadmium exerts its phytotoxicity by interfering with several basic events of plant growth, development and physiology (Qadir *et al*. 2004). Cadmium induces oxidative stress leading to the overproduction of harmful reactive oxygen species (ROS) (Zhang *et al*. 2010). These ROS may cause damage to cell membranes, proteins, DNA replication and repair. A major effect of this metal, observed in most plants studied to date, is the inhibition of photosynthesis by altering chlorophyll synthesis (Pietrini *et al*. 2003; Maksymiec and Krupa 2006) and the light-harvesting Chl *a*/Chl *b* protein complex II (Qureshi *et al*. 2010), and interfering with RuBisCO activation (Prasad 1995). Therefore, Cd-mediated reductions in photosynthetic activity lead to declines in crop yield (Ouzounidou *et al*. 1997). Cadmium also interferes with processes such as carbohydrate and nitrogen metabolism (Sanità di Toppi and Gabbrielli 1999), enzyme catalysis (Van Assche and Clijsters 1990) and water balance (Perfus-Barbeoch *et al*. 2002).

Sulfur plays a critical role in allowing plants to protect themselves against heavy metal toxicity, especially Cd toxicity (Gill and Tuteja 2011). Numerous studies have shown that S is involved in the biosynthesis of heavy metal detoxification agents (Anjum *et al*. 2012), such as non-protein thiols (NPTs) including phytochelatins (PCs), glutathione (GSH) (Noctor *et al*. 2011) and Cd -sulfide crystallites (Robinson *et al*. 1993). Harada *et al*. (2002) reported that Cd induces the production of thiol compounds and transcripts of the S-assimilation pathway. It is noteworthy that a PC-deficient *Arabidopsis thaliana* mutant, cad1, exhibits Cd, As and Zn hypersensitivity (Cobbett and Goldsbrough 2002; Tennstedt *et al*. 2009). Cadmium activates the S-assimilation pathway responsible for the synthesis of cysteine (Cys), a precursor of GSH biosynthesis. Glutathione (an NPT) acts as an important antioxidant in mitigating Cd-induced stress (Meuwly and Rauser 1992). It also plays an important role in PC synthesis, which has a proven role in Cd detoxification (Park *et al*. 2012).

Plants are also provided with an efficient mechanism for protection against ROS and peroxidation reactions. The majority of ROS-scavenging pathways in plants involve superoxide dismutase (SOD), which is found in almost all cellular compartments, the water–water cycle in chloroplasts and the ascorbic acid (AsA)–GSH cycle in chloroplasts, cytosol, mitochondria, apoplast and peroxisomes, in which ascorbate peroxidase (APX) and GR play crucial roles. Catalase (CAT) removes H_2_O_2_ in peroxisomes (Mittler 2002).

A varying degree of antioxidative response and tolerance is exhibited by different plant species. Several studies have demonstrated that most of the main S-responsive genes involved in sulfate assimilation are induced by Cd, suggesting the existence of a general adaptive response to an increase in cellular demand for reduced S (Nocito *et al*. 2006). Hence the availability of S to plants is crucial.

*Brassica juncea* (L.) Czern. (Indian Mustard) belongs to the Brassicaceae family and is an important oilseed crop that is known to tolerate considerable amounts of Cd (Qadir *et al*. 2004). In this study, we investigated the effects of Cd, S-deficiency and their combination on S-assisted defence against oxidative stress, and changes in cellular antioxidant activity, the contents of NPTs, PCs and photosynthetic pigments, and growth parameters in *B. juncea*.

## Methods

### Experimental design

Seeds of mustard (*B. juncea* cv. Pusa Jaikisan), procured from the Indian Agriculture Research Institute (IARI), were treated with 1 % (v/v) sodium hypochlorite solution for 10 min followed by thorough washing in deionized water. The seeds were germinated on moist soil (Soilrite^®^; 1 kg per pot). For S-nutrition, conditions were as described in Abdallah *et al*. (2010). One set of plant cultures, considered as the control, received Hoagland nutrient solution (Hoagland and Arnon 1950) with $300\;\mu M\;\text{S}{{\text{O}}_{4}}^{2-}$ (herein referred to as ‘S-sufficient’) and was designated as (+S). A second set received the same Hoagland nutrient solution with an S concentration 10 times lower ($30\;\mu M\;\text{S}{{\text{O}}_{4}}^{2-},$ herein referred to as ‘S-deficient’) than that of the control, and was designated as (−S). After 10 days of growth, both sets were divided into two further sets. Cadmium (100 µM CdCl_2_) prepared in corresponding Hoagland nutrient media was supplied to one set of +S and −S daily according to the water-holding capacity (WHC) of the soil. The plants were grown in a growth chamber: 16/8 h light/dark period, photon flux density of 150 ± 10 µmol photons m^−2^ s^−1^, 25/20 °C (day/night) temperature and 75 % relative humidity. There were four replicates of each treatment: (*T*_1_ = +S/−Cd), (*T*_2_ = −S/−Cd), (*T*_3_ = −S/+Cd), (*T*_4_ = +S/+Cd). Leaves were harvested from the plants after 7 and 14 days of Cd treatment and immediately used for biochemical parameter, leaf area and also fresh and dry weight (DW) analysis.

### Thiobarbituric acid reactive substances (TBARS)

The magnitude of oxidative stress was measured by estimating the content of TBARS following the method of Heath and Packer (1968). Fresh tissue was ground to a powder using liquid nitrogen in a pre-chilled mortar and pestle. The powder was mixed into a paste in 1 % (w/v) trichloroacetic acid (TCA; 10 mL g^−1^ fresh weight, FW). The extract was centrifuged at 9660 × g for 5 min. A 1.0-mL aliquot of supernatant was placed in a separate tube, to which 4.0 mL of 0.5 % (w/v) thiobarbituric acid (TBA) was added. The mixture was heated at 99 °C for 30 min. It was then quickly cooled in an ice bath and centrifuged at 2817 × g for 5 min to clarify the reaction mixture. The absorbance of the supernatant at 532 nm was measured and corrected for unspecific turbidity by subtracting the value at 600 nm.

### Superoxide dismutase assay

The SOD assay was performed using the method of Dhindsa *et al*. (1981), based on the ability of SOD to inhibit photochemical reduction of nitroblue tetrazolium (NBT). The assay mixture, consisting of 1.5 mL reaction buffer (containing 0.1 M sodium phosphate buffer, pH 7.5, 1 % w/v PVP), 13 mM of l-methionine, 0.1 mL of enzyme extract with equal amounts of 1 M Na_2_CO_3_, 2.25 mM NBT solution, 3 mM EDTA and 60 μM riboflavin and 1.0 mL of double-distilled water (DDW), was incubated under a 15-W fluorescent lamp at 28 °C. The absorbance of the irradiated reaction mixtures at 560 nm was compared with the non-irradiated mixture and per cent inhibition of colour was plotted as a function of the volume of enzyme extract corresponding to 50 % reduction of NTB, which was considered as one unit of enzyme activity and expressed as mg^−1^ protein min^−1^.

### Ascorbate peroxidase assay

The APX extraction and assay were performed as described by Qureshi *et al*. (2007). Frozen leaf (0.3 g) was homogenized in 3 mL cold extraction buffer (0.5 M phosphate buffer containing 1 % (w/v) polyvenylpyrrolidone (PVP), 1 % (v/v) Triton-X 100, 100 mM EDTA, pH 7.8) in an ice bath. The crude extract was centrifuged at 6708 × g for 15 min at 4 °C. The supernatant was used to measure APX activity. All reagents were prepared fresh before the assay. The assay reaction mixture contained 0.1 M potassium phosphate buffer (pH 7.4), 0.5 mM ascorbate, 0.3 % (v/v) H_2_O_2_ and 100 µL enzyme extract in a total volume of 1 mL. The assay was allowed to equilibrate at 25 °C for 1 min before the addition of hydrogen peroxide, which initiated the reaction. Ascorbate peroxidase assay activity was determined by monitoring the rate of ascorbate oxidation as indicated by a reduction in the absorbance at 290 nm for 3 min at 25 °C. A control reaction was prepared by replacing the ascorbate with reaction buffer. A unit of APX is defined as the amount required to oxidizie 1 µmol of ascorbate min^−1^ at 25 °C (290 nm extinction coefficient of 2.8 mmol^−1^ cm^−1^).

### Glutathione reductase assay

To prepare the crude enzyme extract, 0.5 g leaf material was ground to a powder in liquid nitrogen using a pre-chilled mortar and pestle. The powder was homogenized in 2 mL of cold 0.1 M potassium phosphate buffer (pH 7.2) in an ice bath. The crude extract was centrifuged at 5433 × g for 15 min at 4 °C. The supernatant was collected and used for assay. The glutathione reductase (GR) assay was modified from Anderson (1985). The 1-mL assay contained 0.02 mM oxidized glutathione (GSSG) and 0.2 mM NADPH in a buffer (0.1 M potassium phosphate buffer, pH 7.2). The assay was initiated with the addition of 0.2 mL of enzyme extract and activity was monitored by a decrease in absorbance at 340 nm for 3 min at 25 °C. A unit of activity is the amount of enzyme that catalyses the reduction of 1 µmol of GSSG min^−1^ at 25 °C.

### Catalase assay

Catalase (CAT) activity was determined using the method of Aebi (1984). Fresh leaf material (0.5 g), ground in 5 mL of extraction buffer (0.5 M Na phosphate, pH 7.3, 3 mM EDTA, 1 % w/v PVP, 1 % v/v Triton X-100) was centrifuged at 13 148 × g for 20 min at 4 °C. Catalase activity in the supernatant was determined by monitoring the disappearance of H_2_O_2_, according to the decrease in absorbance at 240 nm. The reaction was run in a final volume of 2 mL of reaction buffer (0.5 M Na phosphate, pH 7.3) containing 0.1 mL of 3 mM EDTA, 0.1 mL of enzyme extract and 0.1 mL of 3 mM H_2_O_2_ for 5 min. Catalase activity was calculated by using a coefficient of absorbance of 0.036 mM^−1^ cm^−1^. One unit of enzyme determines the amount necessary to decompose 1 µmol of H_2_O_2_ per min.

### Ascorbate content

Ascorbate (AsA) was estimated using a modification of Law *et al*.'s method (1983). Fresh leaf material (0.1 g) was ground to a powder in a mortar and pestle using liquid nitrogen. The powder was homogenized in 2 mL of extraction buffer and was centrifuged at 6708 × g for 10 min. To 400 µL of supernatant, 10 % (w/v) TCA (200 µL) was added. The mixture was vortex-mixed and cooled in ice for 5 min and 10 µL of 5 M NaOH was added. The supernatant mixture was centrifuged at 822 × g for 5 min.

The supernatant fraction was divided into two separate centrifuge tubes (200 µL each) to measure total (AsA + DHA) and reduced (AsA) ascorbate. To estimate total ascorbate, 100 µL of dithiothreitol (DTT) and 200 µL of reaction buffer (150 mM potassium phosphate buffer) were added and the assay mixture was thoroughly mixed and incubated for 15 min at 25 °C; 100 µL of 0.5 % (w/v) *N*-ethylmaleimide was then added. The contents of the other tube were mixed with 200 µL of reaction buffer (150 mM potassium phosphate buffer) and 200 µL of double-distilled water. Both samples were vortex-mixed and incubated at room temperature for 30 s. To each tube was then added 400 µL of 10 % (w/v) TCA, 400 µL of 44 % (v/v) H_3_PO_4_, 444 µL of 4 % (w/v) bipyridyl and 200 µL of 3 % (w/v) FeCl_3_. After vortex-mixing, samples were incubated at 37 °C for 60 min and the absorbance was recorded at 525 nm on a UV–VIS spectrophotometer (Model DU 640, Beckman, USA). A calibration curve was prepared from different concentrations of AsA. The amount was expressed in nmol g^−1^ FW.

### Glutathione content

Glutathione content was determined using the method of Anderson (1985), which measures total GSH [reduced glutathione (GSH) and oxidized glutathione (GSSG)] using a GR-catalyzed reaction. Glutathione was extracted by homogenizing 0.5 g of frozen fresh leaves in 2 mL of 5 % (w/v) 5-sulfosalicylic acid to reduce the oxidation of GSH. The powder was homogenized in 2 mL of cold 0.5 M potassium phosphate buffer (pH 7.8) in an ice bath. The crude extract was centrifuged at 5433 × g for 15 min at 4 °C and used for subsequent assay.

The 1.0-mL assay for oxidized GSH contained 40 µL of 0.15 % (w/v) 5-5′-dithio-bis(2-nitrobenzoic acid) (DTNB) and 0.5 mL plant extract in 0.1 M potassium phosphate buffer (pH 7.8). The assay mixture (minus GR and leaf extract) was allowed to equilibrate at 30 °C for 5 min. After the addition of GSH-containing extract, the absorbance at 412 nm was monitored. The reaction blank was prepared by replacing the plant extract with 5 % (w/v) 5-sulfosalicylic acid. To the same tube 0.2 units per assay GR from yeast and 50 µL of 0.4 % (w/v) NADPH were added and the reaction was allowed to run for 30 min at 25 °C, after which the absorbance at 412 nm was measured. The concentration of GSH was determined through comparison with a standard curve of the reaction rate as a function of the concentration of GSSG and was expressed as nmol g^−1^ FW.

### Non-protein thiols

Non-protein thiols (NPTs) were estimated with the method described by Howe and Merchant (1992), using Ellman's reagent. A calibration curve was prepared using GSH (Sigma, MO, USA) to estimate NPTs in samples. The amount of NPT was expressed as nmol g^−1^ FW.

### Phytochelatins

Phytochelatin content (PCs) in the tissue was calculated by subtracting the amount of GSH from the amount of total NPTs, and expressed as nmolg^−1^ FW.

$$\text{PCs}\;(\text{nmol}\;{{\text{g}}^{-}}^{1}\;\text{FW})=\text{NPT}-\text{total GSH}.$$

### Chlorophyll estimation and growth parameters

Chlorophyll content was estimated using the method of Hiscox and Israelstam (1979). Fresh leaves were collected, washed with deionized water and kept in vials. Ten millilitres of DMSO were added to the vials, which were then kept in an oven at 65 °C for 1 h. Absorbance was recorded at 480, 645, 520 and 663 nm on a Beckman DU 640B spectrophotometer. The chlorophyll concentration was calculated with the help of formulae given by Arnon (1949) and expressed as mg g^−1^ FW.

Plants were uprooted carefully from the soil, washed with double-distilled water to remove soil and kept between moist filter paper to avoid desiccation. The leaf area was measured with the help of a portable leaf area meter (Model LI 3000A, LI-COR, USA) and expressed in cm^2^ per plant. To determine the leaf dry matter, the plants were dissected at the stem and petiole junction and the leaves were dried in an oven at 65 °C for 2 days. The dried samples were then weighed to determine the plant DW.

### Statistical analysis

All the estimates of sample variability are given in terms of the standard error (SE). A Student's *t-*test was used to identify statistical differences between pairs of means at a confidence level of ≥95 % for each set of data using ANOVA. The data are means ± SE from 10 samples in four replicates (*n* = 4, *P* ≤ 0.05, significant at 5 % level, *P* ≤ 0.01, significant at 1 % level).

## Results

### Thiobarbituric acid reactive substances

**Figure 1.**
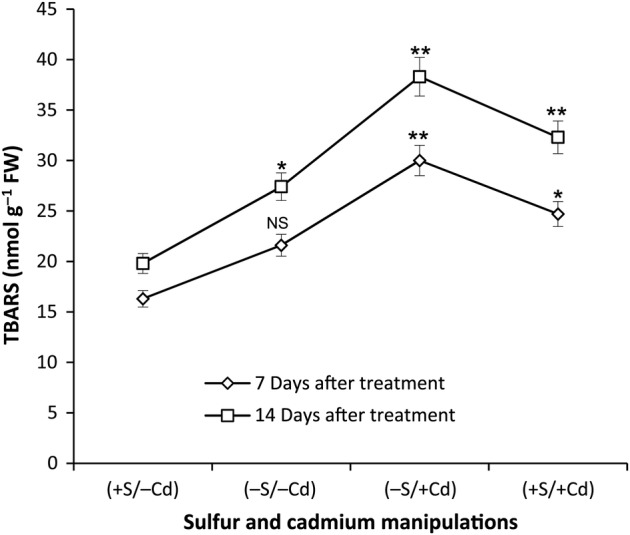
Effect of S-deficiency and Cd stress on the magnitude of oxidative stress in mustard leaf. The values are mean and standard error of the mean (mean ± SE) of 10 samples and four replicates (*n* = 4, *P ≤ 0.05; **P ≤ 0.01; NS, non-significant). +S = 300 µM $\text{S}{{\text{O}}_{4}}^{2-},$ −S = 30 µM $\text{S}{{\text{O}}_{4}}^{2-},$ −Cd = No CdCl_2_, +Cd = 100 µM CdCl_2_.

To assess S-deficiency and Cd-induced oxidative cell damage, the content of TBARS was determined. Under S-deficiency, a 32 % increase in TBARS was observed over the control; a further 84 % increase occurred following the application of Cd to these plants. However, the presence of S (+S/+Cd) lowered the oxidative threat by 33 % at 7 DAT and 32 % at 14 DAT during Cd treatment (Fig. 1).

### Response of enzymatic and non-enzymatic antioxidants

To study the stress response in plants exposed to Cd and the protection offered in the presence of S, changes in the activities of enzymatic and non-enzymatic antioxidants involved in the AsA-GSH cycle were analysed in the leaves of mustard.

### Antioxidative enzymes

**Figure 2.**
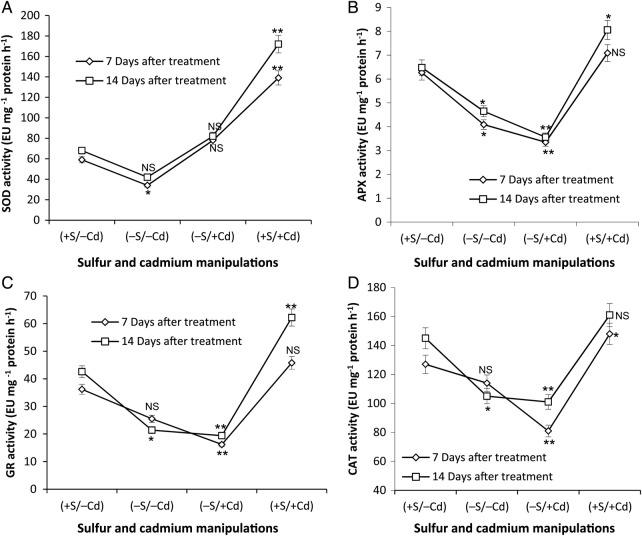
Effect of S-deficiency and Cd stress on the activity of antioxidant enzymes. (A) Superoxide dismutase, (B) APX, (C) GR and (D) catalase (CAT) in mustard leaf. The values are mean and standard error of the mean (mean ± SE) of 10 samples and four replicates (*n* = 4, *P ≤ 0.05; **P ≤ 0.01; NS, non-significant). +S = 300 µM $\text{S}{{\text{O}}_{4}}^{2-},$ −S = 30 µM $\text{S}{{\text{O}}_{4}}^{2-},$ −Cd = No CdCl_2_, +Cd = 100 µM CdCl_2_.

The activity of antioxidative enzymes viz. SOD (Fig. 2A), APX (Fig. 2B), GR (Fig. 2C) and CAT (Fig. 2D) was determined over an experimental period of 7 DAT and 14 DAT of Cd exposure in both S-sufficient and S-deprived plants. Sulfur deficiency significantly suppressed the activities of SOD (42 and 38 %), APX (35 and 28 %), GR (30 and 50 %) and CAT (10 and 28 %) as noted at 7 DAT and 14 DAT, respectively. However, SOD activity was up-regulated in −S/+Cd plants both at 7 DAT (32 %) and 14 DAT (21 %). The activity of SOD further increased to 136 % (7 DAT) and 153 % (14 DAT) under Cd stress when in the presence of sufficient S (+S/+Cd).

Cadmium treatment of S-deprived plants proved the most deleterious, leading to a decline in the activity of APX (47 and 45 %), GR (55 and 54 %) and CAT (36 and 30 %) at 7 DAT and 14 DAT, respectively. However, S-sufficient plants showed significant resistance to Cd stress (+S/+Cd). The activity of APX (13 and 24 %), GR (26 and 46 %) and CAT (16 and 53 %) was increased at 7 DAT and 14 DAT, respectively.

### Non-enzymatic antioxidants

Significant changes in the content of non-enzymatic antioxidants including ascorbates (ascorbate, AsA; dehydroascorbate, DHA; total ascorbate, AsA + DHA) and their ratios (AsA/DHA) were seen under S-deficiency and Cd stress. Sulfur-deprived (−S/−Cd) plants showed a 23 % (7 DAT) and 47 % (14 DAT) increase in DHA content. Cadmium stress during S-deprivation (−S/+Cd) further increased the DHA content by 40 % at 7 DAT and 58 % at 14 DAT. Sulfur-sufficient but Cd-treated (+S/+Cd) plants showed a 33 % (7 DAT) and 60 % (14 DAT) increase in the DHA content.

In contrast to DHA, the AsA content suffered a decline under every treatment and at both time points. AsA declined by 37 and 22 % (−S/−Cd), 42 and 57 % (−S/+Cd) and 33 and 22 % (+S/+Cd) at 7 DAT and 14 DAT, respectively. Similarly, the total ascorbate (AsA + DHA) content suffered a decline, but of a lesser magnitude, in all treatments and at all time points. The total ascorbate content declined by 24 and 10 % (−S/−Cd), 24 and 38 % (−S/+Cd) and 19 and 8 % (+S/+Cd) at 7 DAT and 14 DAT, respectively. The AsA/DHA ratio showed significant alterations; compared with the control (+S/−Cd) value of 3.66 (7 DAT) and 4.95 (14 DAT), the AsA/DHA ratio dropped to 1.87 and 2.63, 1.50 and 1.33 and 1.83 and 2.41 under −S/−Cd, −S/+Cd and +S/+Cd, respectively (Table 1).

**Table 1.** Changes in different attributes of ascorbates (reduced ascorbate, AsA; oxidized ascorbate, DHA; total ascorbate, AsA+DHA and AsA/DHA ratio) under different combinations of Sulfur (S) and Cadmium (Cd) in mustard leaf. Units are expressed in nmol g^−1^ FW. The values are mean and standard error of the mean (mean ± SE) of 10 samples and four replicates (*n* = 4; *P ≤ 0.05; **P ≤ 0.01; NS, non-significant). Figures in parentheses are per cent variations with respect to control (+S/−Cd). +S = 300 µM SO_4_^2−^, −S = 30 µM SO_4_^2−^, −Cd = No CdCl_2_, +Cd = 100 µM CdCl_2_.

Treatment/parameter	Days after treatment (DAT)							
	7				14			
	+S/−Cd	−S/−Cd	−S/+Cd	+S/+Cd	+S/−Cd	−S/−Cd	−S/+Cd	+S/+Cd
	Control				Control			
DHA (nmol g^−1^ FW)	67.3 ± 3.2 (00)	82.9 ± 3.85 (+23 %)^NS^	94.6 ± 4.18 (+40 %)**	89.9 ± 4.17 (+33 %)*	61.6 ± 2.6 (00)	90.6 ± 4.8 (+47 %)**	97.4 ± 5.10 (+58 %)**	98.4 ± 5.33 (+60 %)**
AsA (nmol g^−1^ FW)	246.6 ± 12.2 (00)	155.3 ± 7.5 (−37 %)**	142.3 ± 7.2 (−42 %)**	164.6 ± 7.9 (−33 %)*	304.9 ± 15.3 (00)	238.4 ± 12.7 (−22 %)^NS^	129.4 ± 4.9 (−57 %)**	237.2 ± 9.15 (−22 %)^NS^
AsA + DHA (nmol g^−1^ FW)	313.9 ± 13.4 (00)	238.2 ± 10.6 (−24 %)*	236.9 ± 10.2 (−24 %)*	254.5 ± 12.1 (−19 %)^NS^	366.5 ± 12.3 (00)	329 ± 11.7 (−10 %)^NS^	226.8 ± 9.2 (−38 %)*	335.6 ± 13.1 (−8 %)^NS^
AsA/DHA	3.66	1.87	1.50	1.83	4.95	2.63	1.33	2.41

The GSH contents (reduced form, GSH; oxidized form, GSSG; total GSH, GSH + GSSG) and their ratios (GSH/GSSG) also showed significant alterations. Sulfur-deprived (−S/−Cd) plants showed a decrease of 23 % (7 DAT) and 12 % (14 DAT) in the GSSG content. Cadmium stress during Sulfur deficiency (−S/+Cd) further decreased the GSSG content to 12 % at 7 DAT and 23 % at 14 DAT. Sulfur-sufficient but Cd-treated (+S/+Cd) plants, however, showed a 160 % (7 DAT) and 124 % (14 DAT) increase in the GSSG content.

A similar trend was exhibited by the GSH content in all treatments and at both time points. Glutathione content declined by 28 and 18 % (−S/−Cd), 26 and 34 % (−S/+Cd) at 7 DAT and 14 DAT, respectively. A significant increase of 110 % (7 DAT) and 81 % (14 DAT) in the GSH content over the control was observed in S-sufficient but Cd-treated (+S/+Cd) plants. However, the total GSH content (GSH + GSSG) declined in all treatments, except +S/+Cd, and at both time points. The total GSH content declined by 16 and 17 % (−S/−Cd), 27 and 32 % (−S/+Cd) at 7 DAT and 14 DAT, respectively. However, the presence of sufficient S during Cd stress (+S/+Cd) helped the plant to accumulate 119 % (7 DAT) and 89 % (14 DAT) more total GSH over the control. The GSH/GSSG ratio showed significant alterations; compared with the control (+S/−Cd) value of 4.36 (7 DAT) and 4.50 (14 DAT), the GSH/GSSG ratio dropped to 4.06 and 4.23, 3.66 and 3.81 and 3.52 and 3.63 under −S/−Cd, −S/+Cd and +S/+Cd, respectively (Table 2).

**Table 2.** Changes in different attributes of thiols (−SH) including reduced GSH, oxidized glutathione (GSSG), NPTs and PCs, and soluble protein content under different combinations of Sulfur (S) and Cd in mustard leaf. Values for GSH, GSH and GSH + GSSG are expressed in nmol g^−1^ FW, NPT and PCs in nmol SH mg^−1^ protein and soluble protein in mg g^−1^ FW. The values are the mean and standard error of the mean (mean ± SE) of 10 samples and four replicates (*n* = 4; *P ≤ 0.05; **P ≤ 0.01; NS, non-significant). Numbers in parentheses are per cent variations with respect to control (+S/−Cd). +S = 300 µM SO_4_^2−^, −S = 30 µM SO_4_^2−^, −Cd = No CdCl_2_, +Cd = 100 µM CdCl_2_.

Treatment/parameter	Days after treatment (DAT)							
	7				14			
	+S/−Cd	−S/−Cd	−S/+Cd	+S/+Cd	+S/−Cd	−S/−Cd	−S/+Cd	+S/+Cd
	Control				Control			
GSSG	23.2 ± 1.8 (00)	17.9 ± 1.2 (−23 %)*	20.5 ± 1.4 (−12 %)^NS^	60.4 ± 2.8 (+160 %)**	27.8 ± 1.8 (00)	24.3 ± 1.9 (−12 %)^NS^	21.5 ± 1.8 (−23 %)^NS^	62.4 ± 4.2 (+124 %)**
GSH	101.2 ± 6.1 (00)	72.8 ± 6.2 (−28 %)	75.1 ± 7.1 (−26 %)	212.7 ± 7.2 (+110 %)**	125.2 ± 3.9 (00)	102.8 ± 4.8 (−18 %)^NS^	82.1 ± 3.4 (−34 %)*	226.7 ± 9.8 (+81 %)**
GSH + GSSG	124.4 ± 6.7 (00)	90.7 ± 4.9 (−16 %)^NS^	95.6 ± 5.4 (−27 %)*	273.1 ± 9.6 (+119 %)**	153 ± 4.5 (00)	127.1 ± 5.2 (−17 %)^NS^	103.6 ± 5.2 (−32 %)*	289.1 ± 12.1 (+89 %)**
GSH/GSSG	4.36	4.06	3.66	3.52	4.50	4.23	3.81	3.63
NPTs	445.3 ± 31.3 (00)	155.8 ± 10.1 (−65 %)**	227.8 ± 16.2 (−49 %)*	586.1 ± 36.4 (+32 %)*	495.2 ± 40.2 (00)	154.7 ± 7.4 (−69 %)**	241.6 ± 15.3 (−51 %)*	887.5 ± 100.2 (+79 %)**
PCs	320.9 ± 18.4 (00)	65.1 ± 4.6 (−80 %)**	132.2 ± 9.3 (−59 %)**	313 ± 22.6 (−2 %)^NS^	342.2 ± 24.4 (00)	27.6 ± 2.6 (−92 %)**	138 ± 8.4 (−60 %)**	598.4 ± 44.7 (+75 %)**

### NPTs and PCs

The NPT content declined by 65 and 69 % (−S/−Cd), 49 and 51 % (−S/+Cd) at 7 DAT and 14 DAT, respectively. However, S-sufficient but Cd-treated (+S/+Cd) plants showed a 32 % (7 DAT) and 79 % (14 DAT) increase in the NPT content over the control (Table 2).

Similarly, the PC content (PCs = NPTs − total glutathione) declined under each treatment, except +S/+Cd, at both time points. The PC content declined by 80 and 92 % (−S/−Cd), 59 and 60 % (−S/+Cd) at 7 DAT and 14 DAT, respectively. However, the presence of sufficient S during Cd stress (+S/+Cd) helped the plant to almost maintain (−2 % at 7 DAT) or increase (+75 % at 14 DAT) the PC content over the control (Table 2).

### Chlorophyll and growth

Sulfur deficiency resulted in a significant decrease in chlorophyll content; Chl *a* decreased by 21 % (7 DAT) and 23 % (14 DAT). Moreover, application of Cd to S-deprived (−S/+Cd) plants resulted in a drastic decrease of 53 % at 7 DAT and 55 % at 14 DAT. However, plants with sufficient S were able to resist damage with a decline of 32 and 38 % at 7 DAT and 14 DAT, respectively. The Chl *b* content decreased by 25 % (7 DAT) and 16 % (14 DAT) in −S/−Cd, 39 % (7 DAT) and 39 % (14 DAT) in −S/+Cd and 16 % (7 DAT) and 26 % (14 DAT) in Cd-treated plants supplied with a sufficient amount of S (+S/+Cd). Total Chl (*a* + *b*) content decreased by 22 % (7 DAT) and 21 % (14 DAT) in −S/−Cd, 49 % (7 DAT) and 50 % (14 DAT) in −S/+Cd and, 28 % (7 DAT) and 35 % (14 DAT) in Cd-treated plants supplied with a sufficient amount of S (+S/+Cd). In fact, compared with the control, the ratio of Chl *a* to Chl *b* tended to decrease in plants treated with Cd particularly under Sulfur deficiency (Table 3).

**Table 3.** Changes in chlorophyll contents (mg g^−1^ FW) and chlorophyll a/b ratio in mustard leaf treated with different combinations of Sulfur (S) and Cd. The values are mean and standard error of the mean (mean ± SE) of 10 samples and four replicates (*n* = 4; *P ≤ 0.05; **P ≤ 0.01; NS, non-significant). Numbers in parentheses are per cent variations with respect to control (+S/−Cd). +S = 300 µM SO_4_^2−^, −S = 30 µM SO_4_^2−^, −Cd = No CdCl_2_, +Cd = 100 µM CdCl_2_.

Treatment/parameter	Days after treatment (DAT)							
	7				14			
	+S/−Cd	−S/−Cd	−S/+Cd	+S/+Cd	+S/−Cd	−S/−Cd	−S/+Cd	+S/+Cd
	Control				Control			
Chl *a*	2.38 ± 0.10 (00)	1.87 ± 0.09 (−21 %)^NS^	1.12 ± 0.05 (−53 %)**	1.62 ± 0.07 (−32 %)*	2.52 ± 0.14 (00)	1.93 ± 0.14 (−23 %)*	1.14 ± 0.05 (−55 %)**	1.56 ± 0.06 (−38 %)*
Chl *b*	0.85 ± .04 (00)	0.64 ± 0.03 (−25 %)*	0.52 ± 0.03 (−39 %)*	0.71 ± 0.04 (−16 %)^NS^	0.92 ± 0.06 (00)	0.77 ± 0.05 (−16 %)^NS^	0.56 ± 0.05 (−39 %)*	0.68 ± 0.04 (−26 %)^NS^
Chl *a* + *b*	3.23 ± 0.17 (00)	2.51 ± 0.14 (−22 %)^NS^	1.64 ± 0.10 (−49 %)**	2.33 ± 0.08 (−28 %)*	3.44 ± 0.21 (00)	2.70 ± 0.16 (−21 %)*	1.70 ± 0.12 (−50 %)**	2.24 ± 0.15 (−35 %)*
Chl *a*/*b* ratio	2.80	2.92	2.15	2.28	2.74	2.51	2.03	2.29

**Figure 3.**
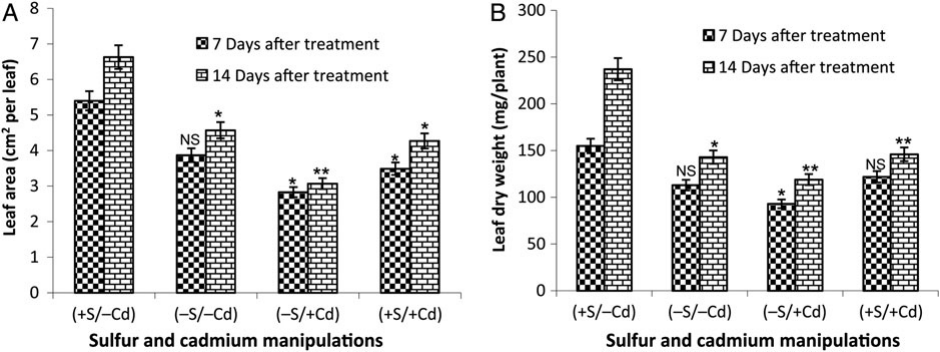
Effect of S-deficiency and Cd stress on (A) leaf area (cm^2^ leaf^−1^) and (B) dry weight (mg plant^−1^) of mustard leaf. The values are mean and standard error of the mean (mean ± SE) of 10 samples and four replicates (*n* = 4; *P ≤ 0.05; **P ≤ 0.01; NS, non-significant). +S = 300 µM $\text{S}{{\text{O}}_{4}}^{2-},$ −S = 30 µM $\text{S}{{\text{O}}_{4}}^{2-},$ −Cd = No CdCl_2_, +Cd = 100 µM CdCl_2_.

Leaf area was also reduced by ∼28 % (7 DAT) and 31 % (14 DAT) in S-deprived plants and the reduction was more pronounced (48 % (7 DAT) and 54 % (14 DAT)) when S-deficient plants were exposed to Cd (Fig. 3A). The presence of S in Cd-stressed plants, however, had a lesser effect of −35 % (7 DAT) and −36 % (14 DAT). Sulfur deficiency led to a reduction of leaf DW by 27 % (7 DAT) and 40 % (14 DAT). Upon exposure to Cd, S-deficient plants further decreased DW, by 40 and 50 % at 7 DAT and 14 DAT, respectively. However, the DW of leaves in S-sufficient plants treated with Cd decreased by 21 % (7 DAT) and 38 % (14 DAT) (Fig. 3B).

## Discussion

Cadmium stress as well as S deprivation leads to oxidative stress in plants (Pilon *et al*. 2006; Bashir *et al*. 2013) due to peroxidation of biomolecules including lipids. In this study, an increase in the magnitude of oxidative stress was observed in response to S-deficiency and Cd stress. Cadmium induced more oxidative stress, particularly in Sulfur deficient conditions. This indicated the formation of more ROS in leaves, not only under S-deficiency but also under Cd stress, with the most formed under dual stress (−S/+Cd). However, the presence of S reduced the magnitude of oxidative stress (Fig. 1), indicating that Cd becomes more deleterious under Sulfur deficiency. This could be because antioxidant systems in plants might suffer a limitation of S for the synthesis of antioxidant enzymes and other peptides/proteins, and ROS quenching molecules such as GSH. Thus, it is evident that Cd severely impairs the plant's ability to counter-attack ROS during S-deficiency. Hu *et al*. (2015) also found that a source of S (SO_2_) helped wheat to resist oxidative stress.

### Antioxidative enzymes

Cadmium influences the ascorbate-GSH antioxidant system (Markovska *et al*. 2009) and metabolism of essential elements (Dong *et al*. 2006), including non-enzymatic (GSH; AsA; α-tocopherol and carotenoids) and enzymatic (SOD, APX, GR, CAT, etc.) antioxidants. In the present study, SOD, APX, GR and CAT activities were studied. The activity of all these enzymes was lower under S-deficiency, which may be attributed to the limitation in the amount of S-containing amino acids and hence lower synthesis of enzymes. Under dual (−S/+Cd) stress the activity of APX, GR and CAT further decreased as a result of S deficiency. Interestingly, Cd increased the activity of SOD despite limited S availability, which perhaps is due to the channelling of infrastructural S towards the synthesis of more SOD, to counter ROS. A similar observation of lesser degree was made in Arabidopsis by Bashir *et al*. (2013). Unlike SOD, APX and GR activities did not increase under dual (−S/+Cd) stress but decreased further, indicating that Cd-tolerant mustard prefers the strengthening of SOD rather than the ascorbate-GSH antioxidant system. These results also clearly show that due to the lower activity of the ascorbate-GSH antioxidant system accumulation of H_2_O_2_ caused high damage to chlorophylls. The maximum decrease in APX, GR and CAT activities occurred under Cd stress during S-deficiency, which could be attributed to the binding of Cd metal with the thiol groups of these enzymes (Romero-Puertas *et al*. 2007). SOD showed a different response perhaps due to Cd entrapment by peptides, GSH/GSH oligomers or antioxidant enzymes, helping up-regulate SOD activity. Sulfur deficiency significantly suppressed APX and GR activities. The decline in APX and GR activities may be due to GSH depletion and a subsequent reduction in the ascorbate-GSH cycle (Gomes-Junior *et al*. 2006) as shown in Fig. 3B and C. The recovery of CAT activity at 14 DAT in S-deficient plants might be due to the presence of comparatively more Fe (metal ligand of CAT) in the absence of S and that the defence system acts better against oxidative stress and/or compensates for the decrease in other antioxidant enzymes such as APX and GR but at non-chloroplast locations in the cell. The scenario was totally different in S-sufficient plants exposed to Cd. The activities of all four enzymes (SOD, APX, GR and CAT) were up-regulated; the maximum SOD was 136–153 %.

### Ascorbate, glutathione and sulfur-rich compounds

The ascorbate-GSH cycle appears to be of great importance in controlling the cellular redox status, especially upon exposure to Cd. Both ascorbate and GSH are essential for scavenging ROS (Anjum *et al*. 2008) and are important in controlling metal homeostasis. The ratio of AsA in the reduced form (AsA) to that in the oxidized state (DHA) is considered an important indicator of the redox status of the cell and the degree of oxidative stress experienced. Data (Table 1) show that there was an overall increase in DHA and a decrease in AsA and total ascorbate content, indicating inhibition of ascorbate synthesis. However, the Asa/DHA ratio varied; from 4.95 (control) it dropped to 2.63 (−S/−Cd), 1.33 (−S/+Cd) and 2.41 (+S/+Cd) at 14 DAT. The AsA/DHA ratios can be attributed to APX activity, which helps in AsA regeneration and increases the AsA/DHA ratio.

Glutathione is the main S-storage compound and an important antioxidant in plants. The content and redox level of GSH was measured to determine the effect of S-deficiency on the amount of GSH. In S-deficient plants, the GSH content decreased compared with the control. This decrease was more pronounced when S-deficient mustard was exposed to Cd. However, when S was supplied to these plants, there was a marked increase in GSH levels (Table 2). Thus, S plays a key role in limiting the cellular damage and inducing defence mechanisms against the ROS in response to Cd treatment through the accumulation of GSH. The combination of Cd stress with S-deficiency provided novel data in the present study, but otherwise the results were consistent with the results obtained by Smeets *et al*. (2005), who reported that heavy metals such as Cd bind to GSH, forming metal-thiolate compounds. This suggests that GSH might be involved in the synthesis of PCs, which could detoxify Cd ions. It also plays an indirect role in protecting membranes by maintaining α-tocopherol and zeaxanthin in the reduced form. Glutathione levels and GSH/GSSG ratios are often indicative of the stress faced by the plant (Tausz *et al*. 2004). The response of GSH and redox state has varied in different studies. In this study, S-deficiency, Cd stress under S-deficiency and Cd stress during sufficient S decreased the GSH/GSSG ratio, from 4.50 in the control to 4.23 (−S/−Cd), 3.81 (−S/+Cd) and 3.63 (+S/+Cd) at 14 DAT, showing a ratio-maintaining capability of plant only in the presence of sufficient S. The data suggest a direct correlation between GR activity and GSH/GSSG ratio.

The addition of sufficient S tended to recover GSH levels and maintain the redox status of the cells during short- and long-term exposure to Cd. Under Cd stress, the formation of Cd–GSH and Cd–PC complexes reduces the free Cd concentration in the cytoplasm and helps suppress activation of the stress-related responses in plant metabolism (Metwally *et al*. 2005). It has been shown that S-nutrition status is associated with plant response to Cd, at least at the plasma membrane H^+^ATPase level (Astolfi *et al*. 2005), with a concomitant uptake of sulfate at a higher rate (Nocito *et al*. 2002). Interestingly, the levels of NPTs and PCs fell far below those of control plants, indicating that the cell utilizes reserves of S-rich compounds that are found in relatively high concentrations in Indian mustard, perhaps making it Cd-tolerant and a hyperaccumulator. Adaptation of sulfate uptake and assimilation is assumed to be a crucial determinant for plant survival in a wide range of adverse environmental conditions since different S-containing compounds are involved in plant responses to both biotic and abiotic stresses (May *et al*. 1998; Rausch and Wachter 2005). Cysteine provided to plants through sulfate assimilation pathways acts as a source of reduced S for the biosynthesis of a number of S-containing compounds, including GSH. Cysteine synthesis has previously been shown to be improved with the addition of S sources, and a Cys deficiency limits the synthesis of GSH (Nikiforova *et al*. 2006; Chekmeneva *et al*. 2011). Therefore, GSH synthesis in plant cells as observed in the present study relies on and is regulated by the plant's S supply. A sufficient amount of S helped plants not only to maintain, but also to accumulate GSH, NPTs and PCs by 90, 79 and 57 %, respectively, over the control at 14 DAT. The level of different types of PCs was shown to increase with 100 µM CdCl_2_ in mustard (D'Allessandro *et al*. 2013) using a proteomic approach, strongly supporting the protective role of PCs against Cd. Phytochelatin binds to toxic metal and then the metal–PC complex is sequestered in the vacuole and perhaps induces specific transporters that mediate Cd tolerance to *Arabidopsis* (Park *et al*. 2012). However, the efficiency of PC-based Cd-detoxification is subject to the availability of other nutrients such as iron (Astolfi *et al*. 2011).

### Chlorophyll and growth

Cadmium may directly inhibit the process of photosynthesis by its interaction with enzymes (Siedlecka and Krupa 1999). Indirectly, Cd may inhibit the synthesis of photosynthetic pigments or cause their degradation; S-deficiency further worsens the scenario (Bashir *et al*. 2013). The effects of Cd on N and S assimilation have been studied in several plants. The current results showed that both Cd and S-deficiency significantly reduced the chlorophyll content and the Chl *a* to Chl *b* ratio in a hyperaccumulator plant, Indian mustard. Both S and Cd, alone or in combination, cause leaf chlorosis (Bashir *et al*. 2013). Several studies have suggested that Cd-induced leaf chlorosis might be due to impairment of the Mg^2+^ insertion into protoporphyrinogen (Gillet *et al*. 2006) or direct Chl destruction as a consequence of Mg ion substitution in both Chl *a* and *b* (Küpper *et al*. 1998). Our results showed clear and very rapid inhibition of Chl *a*, rather than Chl *b*, mainly in response to Cd stress, with or without S-deficiency. As an early response to S-deficiency, the Chl *a/*Chl *b* ratio increased slightly (−S/−Cd at 7 DAT) but in the remaining treatments there was a significant decrease in the Chl *a/*Chl *b* ratio. Cadmium may also impair the S uptake, leading to leaf chlorosis and a disrupted pigment ratio. It has also been proposed that a reduction in chlorophyll might be caused by direct interference by Cd with enzymes of the Chl biosynthesis pathways or by Cd interference with the correct assembly of the pigment–protein complexes of the photosystems (Baryla *et al*. 2001; Qureshi *et al*. 2010). It is also possible that the Chl decrease may be due to strong oxidation of the photochemical apparatus and a reduction in chloroplast density and size. Sulfur deficiency in combination with Cd further alleviated chlorophyll inhibition. However, in the presence of sufficient S, we observed lower destruction of chlorophyll. S is required to repair Fe-S-containing protein complexes, including the incorporation of Fe-S clusters into apoproteins and the stabilization of biomolecules with sulfolipids. It has also been proposed that Cd may influence the biosynthesis of chlorophyll by affecting protochlorophyllide reductase, which, however, contains oxygen-tolerant Fe-S clusters (Nomata *et al*. 2008), indicating the role of S in protection.

When the overall impact of S-deficiency and Cd stress on leaf area and leaf biomass was analysed, S showed a positive role under Cd stress. Sulfur deficiency and Cd caused a significant decrease in leaf area and DW, at least with sufficient S-supply. In the current study, growth inhibition was more severe in −S/+Cd (up to 54 % in leaf area and 50 % in DW) plants than in S-deficiency (up to 31 % in leaf area and 40 % in DW) or Cd stress (up to 36 % in leaf area and 38 % in DW), indicating that the effect of Cd treatment and Sulfur deficiency is synergistic. Moreover, leaf area was found to be more sensitive in response to Cd stress under S-deficiency. Ouzounidou *et al*. (1997) suggested that the inhibitory action of heavy metals on root-shoot and leaf growth seems principally to be due to chromosomal aberrations and abnormal cell divisions. It may also be correlated with Cd-induced inhibition of photosynthetic processes and other enzymes involved in leaf expansion. Decreased plant growth caused by heavy metals is a collective consequence of inhibition of photosynthesis, translocation of photosynthetic products and cell division (Drążkiewicz and Baszyński 2005).

## Conclusions

In conclusion, we confirmed that S-deficiency increases the susceptibility of plants to Cd-generated oxidative damage and modulates the AsA-GSH cycle. We propose that S helps the accumulation of GSH and other S-rich compounds to detoxify metals. In turn, SOD expression is up-regulated to reduce the concentration of superoxide radicals. This study demonstrates that SOD is not the first line of defence against metal stress and that S-rich compounds play a prime role. Further, S-deprived plants lack S-defence and efficient antioxidative mechanisms, making Cd more dangerous. Under S-deficiency, S-containing defence metabolites (GSH, PCs, etc.) as well as enzyme co-factors (Fe-S clusters) decline. The clear decrease in Fe-S clusters, under S-deficiency, is crucial for the maintenance of photosynthesis-related molecules and activities, but the presence of Cd under such conditions might exacerbate oxidative stress, with a highly adverse effect on growth.

## Sources of Funding

The authors extend their appreciation to the Deanship of Scientific Research at King Saud University for funding this work through research group no. RGP-VPP RG-1435-042.

## Contributions by the Authors

The research design and preparation of the manuscript are credited to M.I.Q., H.B. and M.M.I. H.B., R.B., J.A. and M.A.B. contributed to data collection in replicates. M.I.Q., H.B. and I.A.A. analysed the data.

## Conflicts of Interest Statement

None declared.
